# Detection of IL-9 producing T cells in the PBMCs of allergic asthmatic patients

**DOI:** 10.1186/s12865-017-0220-1

**Published:** 2017-07-19

**Authors:** Lei Jia, Ying Wang, Jiangping Li, Sha Li, Yannan Zhang, Juan Shen, Weiping Tan, Changyou Wu

**Affiliations:** 1 Institute of Immunology, Zhongshan School of Medicine, Sun Yat-sen University, Guangzhou, 510080 China; 2 The Third Affiliated Hospital, Sun Yat-sen University, Guangzhou, 510600 China; 3 Department of Pediatrics, Sun Yat-sen Memorial Hospital, Sun Yat-sen University, Guangzhou, 510120 China

**Keywords:** IL-9, Th9 cell, IgE, Allergic asthma

## Abstract

**Background:**

Interleukin-9 (IL-9) was reported as an active participant in the pathogenesis of allergic asthma. This study aimed to investigate the major source ofIL-9 and its effect on interferon γ (IFN-γ) and immunoglobulin (Ig) secretion by B cells.

**Methods:**

We isolated peripheral blood mononuclear cells from children with allergic asthma and healthy children. IL-9, IL-4 and IFN-γ expression were detected by ELISA, ELISpot and Flowcytometry. Expression of transcription factor PU.1 was measured by Western Blot. We evaluated the effect of IL-9 on B cell activation and Ig production.

**Results:**

Results showed that compared with healthy children, levels of IL-9, IL-4 and PU.1 were elevated and levels of IFN-γ were lower in children with allergic asthma. IL-9-expressing CD4^+^ T cells did not co-express IL-4. Exogenous IL-9 inhibited IFN-γ production in a dose-dependent manner. Antigen-specific Th9 cells existed in children with house dust mite allergic asthma. IL-9 up-regulated expression of CD69 and CD25 on B cells and combination of IL-9 and IL-4 enhanced IgE production.

**Conclusions:**

In conclusion, our results showed that Th9 cells may be the major source of IL-9 in children with allergic asthma. In these patients, IL-9 impairs IFN-γ production and synergistically promotes IL-4-induced IgE secretion.

## Background

Asthma is a chronic inflammatory respiratory disease, of which allergic asthma is the most common phenotype [1, 2]. It often occurs in childhood and is associated with allergy. Immune response plays a significant role in the pathogenesis of allergic asthma. T helper (Th) cells and their lineage-specific cytokines regulate the immune response of allergic airway inflammation that results in airway hyper-responsiveness [3]. Th2 cells and their lineage-specific cytokines have been documented as regulators of allergic immunity [4–6]. High concentration of interleukin-4 (IL-4) in the bronchoalveolar lavage (BAL) fluid induces Ig class switching synthesized by B cells to generate abundant of immunoglobulin E (IgE). IL-5, another important Th2 effector cytokine, promotes the development of eosinophil in bone marrow and eosinophilia in lung tissue. Th2 cells also cause goblet cell metaplasia and bronchial hyperrectivity, as well as structural changes in the vessel wall by up-regulation of the adhesion molecular vascular cell adhesion protein 1(VCAM-1) and intercellular adhesion molecule 1 (ICAM-1)via production of IL-13 [7, 8]. These changes facilitate the exit of eosinophils.

In addition to Th2 cells and their effector cytokines, recent studies showed that IL-9 is also implicated [9, 10]. IL-9 was originally considered as a member of Th2 cell cytokines induced by IL-2, IL-4 and transforming growth factor (TGF-β), and was repressed by interferon γ (IFN-γ) [11, 12]. However, studies using various mouse models, includingIL-9-transgenic-, IL-9^−/−^, IL-9R^−/−^ mice, and IL-9-fluorescent reporter mice, have shown that Th9 cells, instead of Th2 or innate lymphoid cells (ILCs), represents the major source of IL-9 [12, 13]. In vitro studies have also reported that IL-9 and IL-4 cannot be produced by the same subtype of T cells, indicating that Th9 cells are a distinct subset of Th cells [14].

Th9 cells were initially defined by Veldhoen et al. and Dardalhon et al. in 2008 [14, 15]. Th9 cell is a distinct Th subset because of lack of other subsets’ transcription factors like T-bet for Th1 cell, GATA-3 for Th2 cell, RORγt for Th17 cell and Foxp3 for Treg cell. IL-4 plus TGF-β are the critical polarizing factors for Th9 differentiation [16]. However, IL-9 production by IL-4 knockout Th cells implies that there is an IL-4-independent pathway. STAT-6, IRF4, and GATA-3 are the major transcription factors for Th9 cell development under IL-4 condition [14, 15, 17, 18].The transduction of TGF-β signal in Th9 cell relies on Smad activation and PU.1 expression. IL-9 is over-expressed in BAL fluid and lung tissue in asthma patients and the quantity of Th9 cells directly correlated with AHR severity, whereas IL-9 neutralization relieves local inflammation [19–21]. IL-9 can up-regulate antibodies secretion by IL-4-stimulated B cells and promotes goblet cell metaplasia [22–24]. Considering the important role of IL-9 that may plays in the immune response, determining the major source of IL-9 will further increase our understanding of the pathogenesis of allergic asthma.

This study aimed to investigate the major source of IL-9 and its effect on interferon γ (IFN-γ) and immunoglobulin (Ig) secretion by B cells. Our results showed that peripheral blood mononuclear cells (PBMCs) from children with allergic asthma produced higher levels of IL-9 and IL-4 and lower levels of IFN-γ than healthy children. Intracellular staining showed that IL-9-expressing CD4^+^ T cells did not co-express IL-4. Results also showed higher production of transcription factor PU.1 and house dust mite (HDM)-specific IL-9-expressing cells, indicating that Th9 cells but not Th2 cells are the major source of IL-9 in allergic asthma. Functional analysis showed that exogenous IL-9 inhibited IFN-γ production in a dose-dependent manner, up-regulated B cell activation, and enhancedIL-4-driven IgG and IgE secretion by B cells.

## Methods

### Patients

Ten healthy children and twenty-nine children with allergic asthma were recruited from Department of Pediatrics, Sun Yat-sen Memorial Hospital, SunYat-sen University (Table 1). Asthmatic patients were given diagnoses according to Global Initiative for Asthma guidelines. For children 6–16-years-old, asthma was defined as the presence of relevant clinical history (i.e. history of recurrent dyspnea, wheezing or cough episodes) and pulmonary function tests demonstrating variable airflow limitation by means of airway responsiveness testing or airway reversibility testing. For children under 5 years old, asthma was defined as the presence of relevant clinical history with support of therapeutic trial or tests for atopy. Inclusion criteria for asthmatic patients were defined as having allergic asthma when specific IgE can be detected in the serum together with wheeze induced primarily by allergens but not respiratory tract infections. Exclusion criteria include other chronic, pulmonary and autoimmune diseases; immunodeficiency; ingestion of steroids, antibiotics or probiotics; and infection14 days prior to blood donation. Healthy controls included healthy children without any clinical allergic symptoms. Informed consents were obtained from legal guardians of these children. All the experiments were approved by the Ethical Committee of Sun Yat-sen Memorial Hospital and Zhongshan School of Medicine, Sun Yat-sen University.

**Table 1:** Subjects’ characteristics

Characteristics	Healthy children (*n* = 10)	Children with allergic asthma (*n* = 29)
Age (years), mean (range)	5.15 (3.5–10)	5.58 (3.1–13)
Gender (male/female)	8/2	22/7
Specific allergen IgE positive	–	29
A single House dust mite		9
Mutiple allergen (including dust mite and other allergens )		10
Pollen		5
Cat dander and/or dog dander		3
Fungus		2

### Antibodies and reagents

For cell phenotypic and intracellular cytokines study and transcription factor analysis, FITC-labeled anti-CD3, APC-Cy7-labelled anti-CD4, PE labeled anti-CD19, FITC-labeled anti-CD25, PE-Cy7-labelled anti-CD69, PE-Cy7-labelled anti-IFN-γ, APC-labeled anti-IL-4, PE-labeled anti-IL-9 were obtained from BD Biosciences (San Jose, CA, US). For Western blotting, mouse anti-PU.1 mAb was purchased from Cell Signaling Technology (Danvers, MA, US). Mouse anti-β-actin and anti-mouse IgG-HRP were bought from Santa Cruz Biotechnology (Santa Cruz, CA, US). Human ELSIA kits for cytokine IFN-γ, IL-4, IL-9, IgG and IgE were purchased from BD Biosciences. ELISpot kits for cytokine IFN-γ, IL-4 were purchased from BD Biosciences. IL-9 ELISpot Kit was produced by R&D Systems (Minneapolis, MN, US). Purified anti-CD3, anti-CD28 and anti-CD40 monoclonal antibodies (mAbs) were from the production of BD Biosciences. Recombination IL-9 and IL-4 were from Peprotech (Rocky Hill, NJ, US). House dust mite extract was purchased from GREER Company (Lenoir, NC, US).PMA, ionomycin and Brefeldin A were produced by Sigma-Aldrich (St. Louis, US).

### Cell isolation and culture

PBMCs were isolated from heparinized blood within 24 h of blood drawing using Ficoll–Hypaque gradient (Haoyang Biological Manufacture, Tianjin, China) centrifugation and were washed twice in Hanks’ balanced salt solution. CD4^+^ T cells were positively separated with anti-CD4-conjugated magnetic MACS microbeads (Miltenyi, Germany). The purity of CD4^+^ T cells evaluated by flowcytometry analysis and was more than 97%. Cells were suspended at a concentration of 2 × 10^6^/ml in complete RPMI 1640 medium (Gibco, Grand Island, NY, US) supplemented with 10% FCS (Sijiqing, China), 100 U/ml penicillin, 100 μg/ml streptomycin, 50 μM 2-mercaptoethanol and 2 mM L-glutamine (all from Gibco BRL). B cells were positively separated with anti-CD19-conjugated magnetic MACS microbeads (Miltenyi, Germany). The purity of B cells was >98%, as assessed by flowcytometry analysis. Then B cells were cultured at a concentration of 10^5^ cells/ml with plate-coated anti-CD40 with different combination of IL-4 andIL-9 in complete RPMI1640 for 10 days.

### ELISA and ELISpot

Cells were cultured in complete RPMI 1640 for 48 h with PMA (20 ng/ml) plus ionomycin (1 μg/ml), or plated-coated anti-CD3 (1 μg/ml) with or without IL-9, or HDM extract (5, 10, 20, and 40 μg/ml), or anti-CD40 in the presence or absence of IL-9 or IL-4 in a round-bottom 96-well plate at a concentration of 2 × 10^6^ cells/ml in triplicate and incubated at 37 °C with 5%CO_2_. The supernatants were harvested, and concentrations of cytokine IFN-γ, IL-9, IL-4, IgE and IgG were detected by ELISA. The detection limits of IFN-γ, IL-9, IL-4, IgE and IgG assay kits were 4.7 pg/ml, 1 pg/ml, 4.7 pg/ml, 7.8 ng/ml and 1.6 ng/ml, respectively. Cells were suspended in complete RPMI-1640 medium and cultured for 20 h with PMA plus ionomycinin a 96-well plate at a concentration of 2 × 10^6^ cells/ml in triplicate and incubated at 37 °C with 5%CO_2_. The secretion of IFN-γ, IL-9 and IL-4 were detected by ELISpot.

### Cell surface and intracellular staining analysis

For flowcytometry analysis, cells were stimulated with 20 ng/ml PMA plus 1 μg/ml ionomycin or HDM extract 20 μg/ml together with 10 μg/ml Brefeldin A. For surface marker staining, cells were incubated with respective fluorescent-labeled antibodies at 4 °C in dark for 30 min, washed with PBS twice and fixed in 0.5% paraformaldehyde before acquisition. For intracellular cytokine staining, stimulated cells were fixed in 4% paraformaldehyde, followed by permeabilization and staining in PBS containing 0.1% saponin and then stained with respective fluorescent-labeled antibodies. These cells were acquired by Aria II (Becton Dickinson, San Jose, CA, US) and analyzed by FlowJo software (Treestar, San Carlos, CA, US).

### Western blotting

PBMCs from healthy children and children with allergic asthma without stimulation were lysed. Lysates were resolved in 10% SDS gels. Protein was transferred into PVDF membranes and immunoblotted with anti-β-actin, and PU.1 mAbs for more than 2 h at room temperature. Membranes were then washed with 1 × PBS twice, and immunoblotted with anti-mouse-HRP for 1 h at room temperature.

### Data analysis

Data were expressed as Mean ± SEM. Independent two-tailed Student’s *t*-test was used for between-group comparisons. Analysis was performed using GraphPad Prism (GraphPadInc, La Jolla, CA, US).

## Results

### Production of IL-9, IL-4 and IFN-γ by PBMCs in children with allergic asthma

**Fig. 1:**
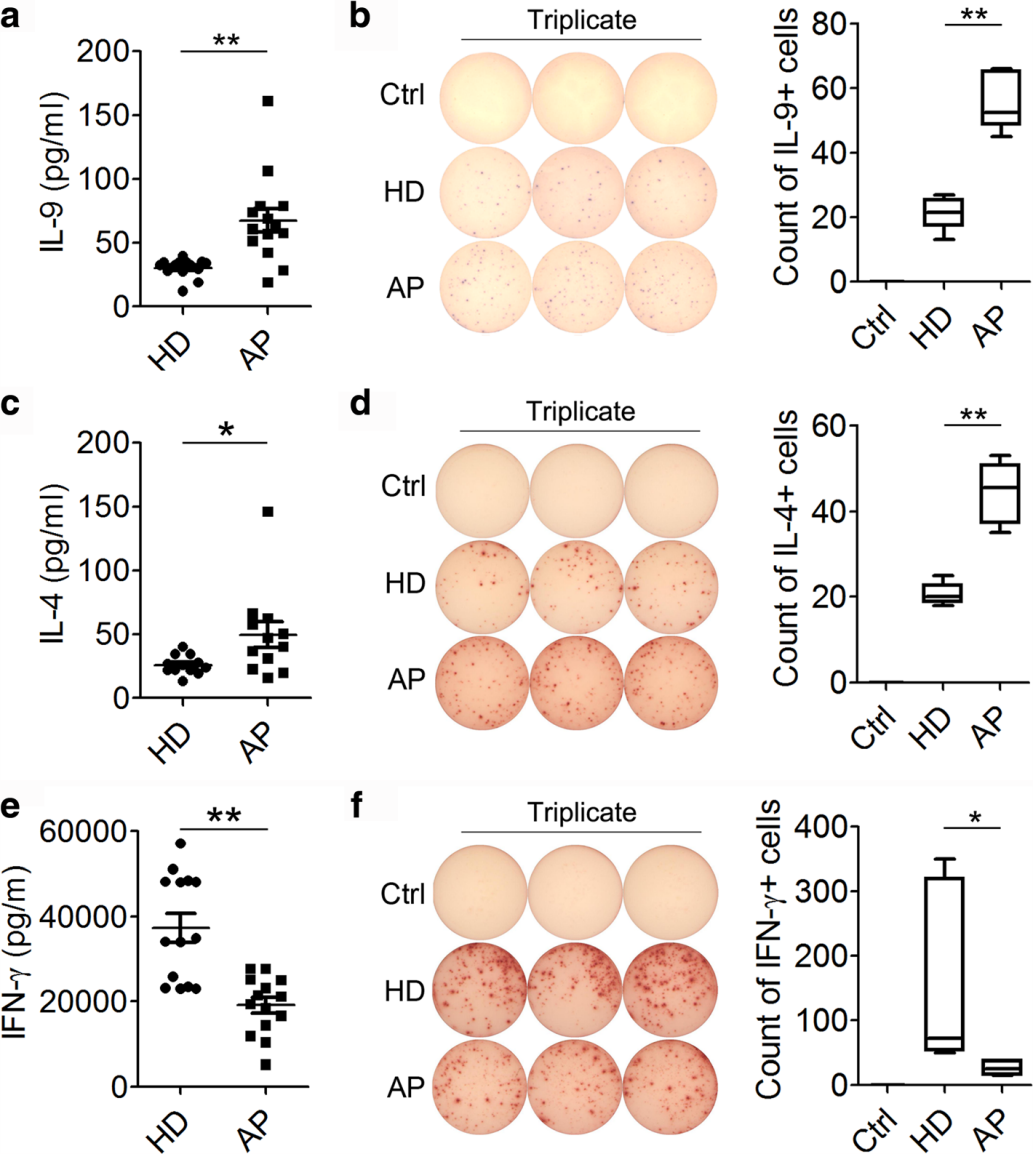
IL-9, IL-4 and IFN-γ production by PBMCs from children with allergic asthma. IL-9 (**a**), IL-4 (**c**) and IFN-γ (**e**) production by PBMCs from PBMCs of healthy donors (HD) and allergic asthma patients (AP) was detected by ELISA. Representative and statistical ELISpot data of IL-9 (**b**), IL-4 (**d**) and IFN-γ (**f**) were obtained from 3 independent experiments. ELISA data were collected from 10 healthy donors and 14 asthma patients. *, *P* < 0.05; **, *P* < 0.01; ***, *P* < 0.001

We first detected the production of IL-9 and IL-4, and IFN-γ by PBMCs from children with allergic asthma compared with healthy children of similar age. IFN-γ is the most important effector cytokine of Th1 cells, which has been reported to inhibit the differentiation of Th2 cell. Cells were stimulated with polyclonal stimulants PMA plus ionomycin for 48 h, and then the concentrations of IL-9, IL-4 and IFN-γ in the supernatants were detected by ELISA. Levels of IL-9 and IL-4 in children with allergic asthma(30.23 ± 1.92 pg/ml and 25.81 ± 2.14 pg/ml, respectively) were significantly higher than those in healthy children (67.37 ± 9.27 pg/ml and 49.50 ± 9.99 pg/ml, respectively) (Fig. 1a and c). On the contrary, levels of IFN-γ in children with allergic asthma were significantly reduced (19.10 ± 1.81 ng/ml vs. 37.29 ± 3.33 ng/ml) (Fig. 1e). ELISpot showed similar results with those of ELISA (Fig. 1b–f).

### Exogenous IL-9 inhibitsIFN-γ production

**Fig. 2:**
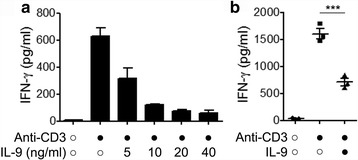
IL-9 inhibits the production of IFN-γ. IL-9 inhibits IFN-γ production from PBMCs in a dose-dependent manner (**a**). IFN-γ production by purified CD4^+^ T cells was detected by ELISA (**b**). ELISA data were collected from 3 independent experiments. ***,*P* < 0.001

In view of the reduced IFN-γ levels and increased IL-9 levels by PBMCs from children with allergic asthma, we investigated the function of IL-9 on the production of IFN-γ by Th1 cells. Cells were stimulated with plate-coated anti-CD3, and then different concentrations of exogenous human IL-9 were added to the cultures. In the absence of IL-9, PBMCs produced 631.14 ± 83.99 pg/ml of IFN-γ. When different doses of IL-9 (5 ng/ml, 10 ng/ml, 20 ng/ml, and 40 ng/ml) were added, levels of IFN-γ in the supernatants were significantly reduced (317.72 ± 103.89 pg/ml, 119.72 ± 9.67 pg/ml, 73.89 ± 16.49 pg/ml, and 61.15 ± 26.54 pg/ml, respectively) (Fig. 2a). Purified CD4^+^ T cells were separated from PBMCs of children with allergic asthma, and were stimulated with plate-coated anti-CD3 in the presence or absence of 20 ng/ml IL-9 for 3 days, the concentration of IFN-γ in the supernatants were then detected by ELISA (Fig. 2b). The results showed that IL-9 significantly down-regulated IFN-γ production by CD4^+^ T cells in a dose-dependent manner.

### Th9 cells are the major source of IL-9 in children with allergic asthma

**Fig. 3:**
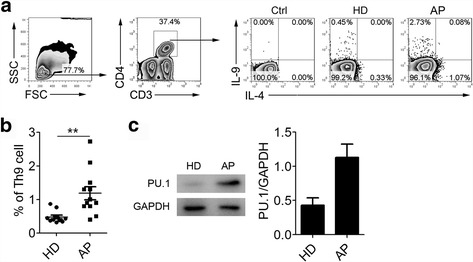
The major IL-9-expressing CD4^+^ T cells in children with allergic asthma are Th9 cells. The percentage of Th9 cells in PBMCs of healthy donors and asthma patients was detected by FACS (**a**). Statistical result was obtained from HD (*n* = 10) and AP (*n* = 12) (**b**). PU.1 levels in total HD and AP PBMCs were measured by Western Blot (**c**). **, *P* < 0.01

Both IL-9 and IL-4 were originally considered as effector cytokines of Th2 cells. However, it appears that there are different signaling pathways and transcription factors that orchestrate the differentiation of IL-9-expressing CD4^+^ T cells [12, 13]. After stimulation with PMA plus ionomycin in the presence of BFA, intracellular staining showed that PBMCs from children with allergic asthma expressed higher level of IL-9. In addition, IL-9-expressing CD4^+^ T cells did not co-express IL-4, indicating that large percentage of IL-9-expressing CD4^+^ T cells in children with allergic asthma might be Th9 cells in origin, instead of Th2 cells (Fig. 3a and b). To confirm this, we detected the major transcription factor of PU.1. It’s well known that TGF-β-induced expression of PU.1 is indispensable for the production of IL-9 [25–27].Over-expression of PU.1 increases IL-9 production and decreases IL-4 production. On the contrary, down-regulation of PU.1 expression in CD4^+^ T cells results in lowered IL-9 production and increases the population of IL-4-expressing Th2 cells. Results from Western blot showed that PU.1 protein was significantly higher in PBMCs from children with allergic asthma than that from healthy children (Fig. 3c). This finding indicates that Th9 cells are the major source of IL-9 in children with allergic asthma.

### House dust mite (HDM) antigen-specific Th9 cells existed in children with allergic asthma

**Fig. 4:**
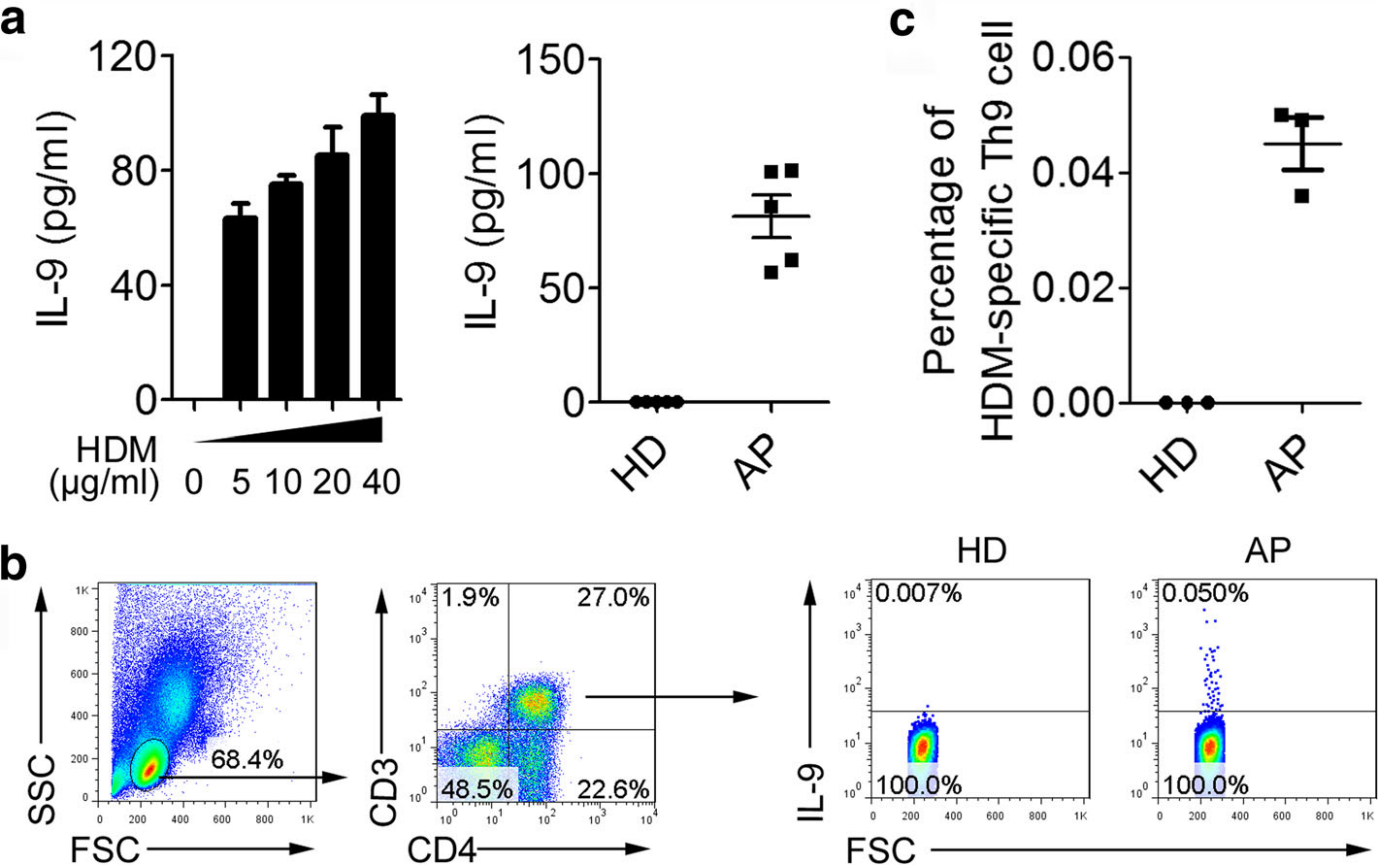
HDM-specific Th9 cells in children with allergic asthma. **a** The production of IL-9 by PBMCs of children with house dust mite allergic asthma (*n* = 5). PBMCs were stimulated with house dust mite (HDM) extract and IL-9 was detected by ELISA. **b** Representative FACS data of HDM-specific Th9 cell in children with HDM allergic asthma. **c** Statistical result of HDM-specific Th9 cell in children with HDM allergic asthma (*n* = 3)

Most of children with allergic asthma are allergic to the (lipo) protein by inhalation or ingestion, such as HDM, animal dander, fungal spores, pollen or peanuts [3, 28]. To investigate the characteristics of antigen-specific Th9 cells in children with allergic asthma, we selected children who were specifically allergic to HDM. PBMCs were isolated from whole blood in these patients. PBMCs were stimulated with different concentrations (0 μg/ml, 5 μg/ml, 10 μg/ml, 20 μg/ml, 40 μg/ml) of HDM extract, and the levels of IL-9 in the supernatants were detected by ELISA. Results showed that HDM extract increased the secretion of IL-9 in a dose-dependent manner in vitro. Then isolated PBMCs from 5 healthy children and 5 children with allergic asthma were stimulated with 10 μg/ml HDM extract at the concentration of 2 × 10^6^cells/ml for 48 h.IL-9 levels in the supernatants were tested by ELISA. Compared with healthy children, HDM extract induced higher levels of IL-9 production in children with allergic asthma (Fig. 4a). Intracellular staining analysis further showed that HDM induced high percentages of IL-9-expressing CD4^+^ T cells (0.045 ± 0.008%) in children with allergic asthma, but not in healthy children (Fig. 4b and c).

### IL-9 activates B cell and promotes IL-4-induced IgE production

**Fig. 5:**
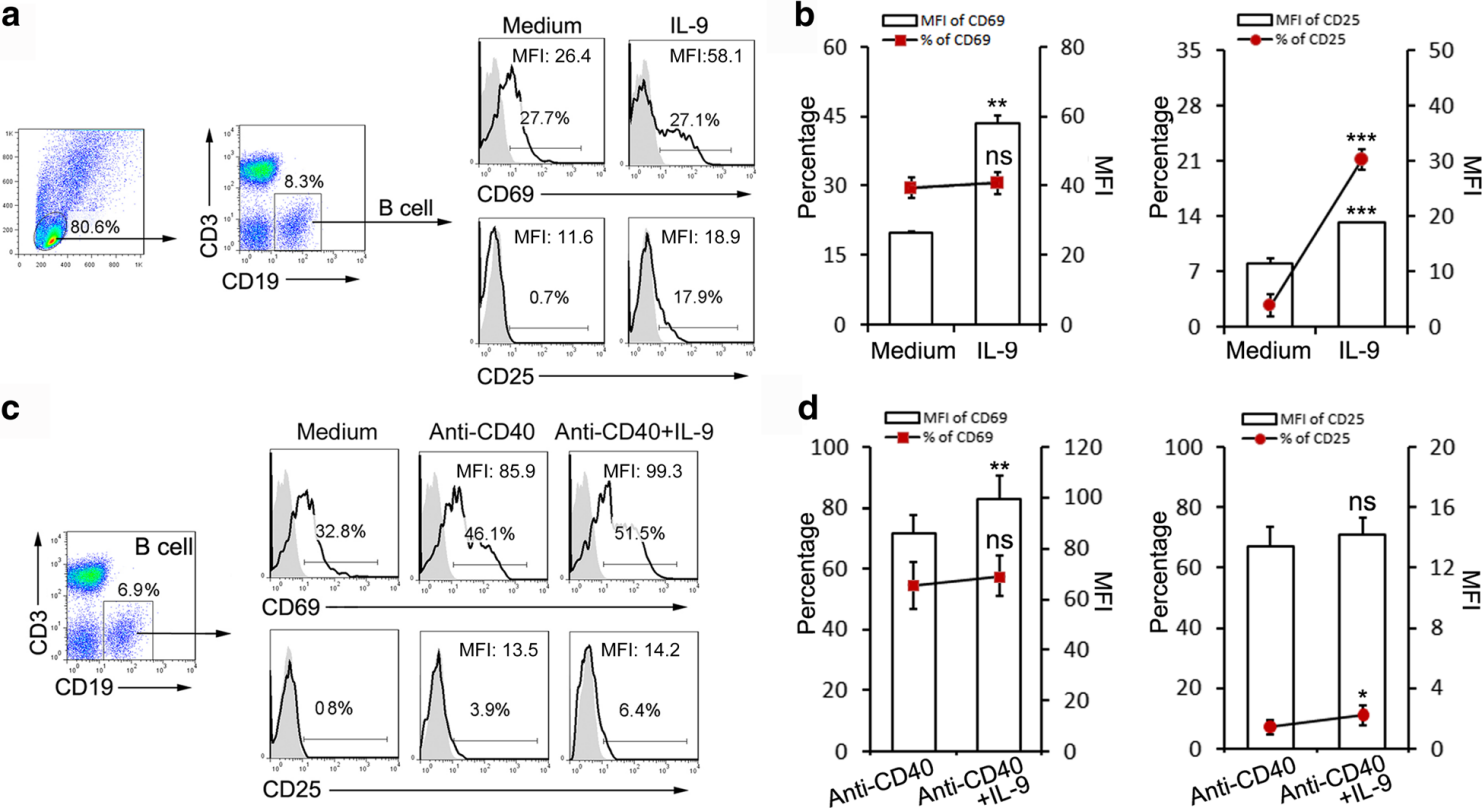
IL-9 promotes B cells activation. **a** Representative FACS data of IL-9 activation function on B cells. **b** Statistical results of IL-9 activation function on B cells. IL-9, alone, up-regulates the Mean Fluorescence Intensity (MFI) of CD69 and CD25 and increases the percentage of CD25 (*n* = 6). **c** Representative FACS data of synergistic effect of IL-9 on B cell activation. **d** Statistical results of IL-9 synergistic activation function on B cells. IL-9, synergistically with Anti-CD40, up-regulates the MFI of CD69 and increases the percentage of CD25 (*n* = 10). ns, *P >* 0.05; *, *P* < 0.05; **, *P* < 0.01; ***, *P* < 0.001

**Fig. 6:**
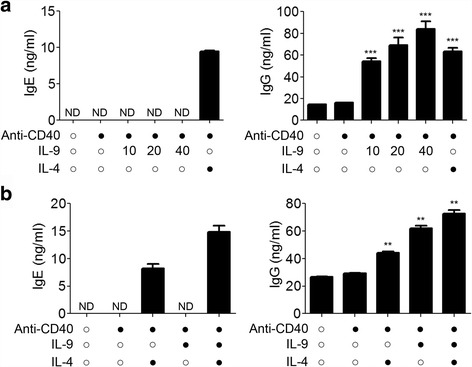
IL-9 synergistically promotes IL-4-induced IgE and IgG production. **a** The production of IgG, but not IgE, can be promoted by IL-9 alone (*n* = 3). **b** Synergistic effect of IL-9 on IL-4–induced IgE and IgG production (*n* = 4). IgE and IgG levels were detected by ELISA. Results represent the Mean ± SEM. ND, not detected; **, *P* < 0.01; ***, *P* < 0.001

The presence of serum IgE (atopy) driven by sensitized B cells is the hallmark of adaptive Th2 cell immunity in patients with allergic asthma [3]. High level of IL-4 in these patients’ serum contributes to the class switching of the immunoglobulins. IL-9 is considered as a mast cell growth factor that increases antibodies secretion by B cells after IL-4 simulation. Our results showed that co-culture with exogenous IL-9 for 3 days, percentages of CD25 expression on B cells was markedly increased and mean fluorescence intensity (MFI) of CD69 and CD25 on activated B cells were increased by IL-9 (Fig. 5a and b). Co-stimulatory molecule anti-CD40 was used to activate B cells, and increase the expression of CD25 and CD69. We also observed increase of CD25 and CD69 expression on B cells. When exogenous IL-9 was added into the culture, MFI or percentage of CD25 and CD69 on B cells were significantly increased (Fig. 5c and d). In addition, anti-CD40 alone did not induce production of IgE and IgG, while combination of anti-CD40 and IL-9 induced significant production of IgG, but not IgE, in a dose-dependent manner. Co-stimulation of IL-4 and anti-CD40, however, induced both IgE and IgG secretion (Fig. 6a). IL-9 alone did not induce the production of IgE. Instead, IL-9 enhanced IL-4-induced IgE and IgG production. Combining exogenous IL-9, IL-4 and anti-CD40 significantly enhanced IgG and IgE secretion by B cells (Fig. 6b).

## Discussion

Asthma is a common chronic inflammatory disease that affects more than 300 million people worldwide. In developed countries, nearly1 in 10 children and 1 in 12 adults is affected [2, 3]. Allergic asthma is the major phenotypes of asthma. Childhood allergic asthma is usually associated with family history of allergies and/or allergic diseases, such as eczema, allergic rhinitis or food or drug allergy. Patients with allergic asthma often have IgE reactivity to allergens in the serum. Histopathology involves infiltration of eosinophils and mast cells, as well as the disorder of Th cell subsets in lung tissue.

In this study, we found that PBMCs from children with allergic asthma produced and secreted higher levels of Th2-specific cytokines IL-4, higher levels of IL-9, and lower levels of Th1-specificcytokine IFN-γ compared to healthy children. When analyses were restricted to children allergic to HDM, intracellular staining detected HDM allergen-specific IL-9-expressing CD4^+^ T cells. HDM allergens induced IL-9 production in a dose-dependent manner. IL-9 was originally described as a Th2-derived cytokine. However, recent studies by Veldhoen et al. suggested that IL-9-producing CD4^+^ T cells belong to a distinct helper T cell subset [14]. Our intracellular staining analysis also indicated that IL-9 was marked expressed by CD4^+^ T cells, and IL-9-positive CD4^+^ T cells did not co-expressTh2-related cytokine IL-4. Together, these findings indicate that high level of IL-9 in children with allergic asthma may be produced by Th9 cells, instead of Th2 cells.

We further found that transcription factor PU.1 was also up-regulated in children with allergic asthma. PU.1 is considered one of the most important and specific transcription factor for Th9 cell differentiation, but not Th2 cell development.PU.1 is indispensable for IL-9 production. In PU.1-deficient mice model, up-regulation of IRF4 binding to *Il10* locus, up-regulation of GATA-3 binding to *Il4* locus have been observed, which indicates potential functions of PU.1 to modify the functions of Th2-related transcription factors and to interfere the generation of Th2 cell-related cytokines [27, 29, 30]. On the contrary, PU.1 directly binds to *Il9* locus and initiates the downstream genetic modification to enhance the production of IL-9 [26, 27].

Up-regulation of IL-9 production and down-regulation of IFN-γ production by mononuclear cells were detected in children with allergic asthma. IL-9 promotes inflammatory response in which Th2 cells are also involved. In the ovalbumin (OVA)-induced inflammatory mice model, neutralization of IL-9 is beneficial to reverse several inflammatory features, such as airway hyper-responsiveness, recruitment of eosinophils, and metaplasia of goblet cells [26, 31]. In PU.1-dificient mice, absence of Th9 cell differentiation and IL-9 production reduce the outbreak of airway inflammation, recruitment of inflammatory cells, and airway remodeling. However, the effect of IL-9 on Th1 cell-mediated IFN-γ production is still not clear. In general, IFN-γ is considered as an inhibitory cytokine of IL-9 generation and Th9 cell differentiation. Down-regulation of IFN-γ in allergic asthma has been reported in previous studies [32, 33].We also observed that the production and secretion of IFN-γ were decreased in children with allergic asthma. Furthermore, our results demonstrated that exogenous IL-9 inhibited IFN-γ production by PBMCs or purified CD4^+^ T cells from children with allergic asthma in a dose-dependent manner.

The presence of high serum titers of atopy IgE in serum is the hallmark of allergic asthma immunity.Th2 cell effector cytokine IL-4 contributes to plasma class switching of the immunoglobulins in B cells. IL-9 is a mast cell growth and differentiation factor.IL-9 has been reported to promote IL-4-driven antibody production by B cells, as well as to up-regulate IL-6 production [12, 23]. However, the direct effect of IL-9 on modulating Ig secretion is not well known. In this study, we found that exogenous IL-9 induced the expression of CD69 and CD25 on B cells, and co-stimulatory signal pathway CD40-CD40L enhanced the effect of IL-9 on B cell activation. In addition, IL-9 induced the production of IgG in a dose-dependent manner in the presence of anti-CD40. IL-9 up-regulates the effect of IL-4 on inducing IgE secretion in the presence of anti-CD40, even though IL-9 alone doesn’t induce the production of IgE. IL-9 might contribute to immunoglobulin (Ig) class switching, but not Ig secretion. More studies are required to investigate the effect and mechanism of IL-9 on immunoglobulin class switching.

In conclusion, our studies showed that high level of IL-9 in children with allergic asthma was mainly produced by Th9 cells, instead of Th2 cells. Transcription factor PU.1 was associated with IL-9 production. Functional analysis further showed that IL-9 directly inhibited IFN-γ production. IL-9 also activated B cells, induced IgG secretion in a dose-dependent manner in the presence anti-CD40. Combination of IL-9, IL-4 and anti-CD40 enhanced IgE secretion.

## Conclusion

In our present work, we demonstrate that IL-9, which is mainly produced by Th9 cells in children with allergic asthma, impairs IFN-γ production and synergistically promotes IL-4-induced IgE secretion.

## Abbreviations

AP: Asthma patient
BAL: Bronchoalveolarlavage
CD40L: CD40 ligand
ELISA: Enzyme-linked immunosorbent assay
ELISpot: Enzyme-linkedimmunospotassay
FACS: Fluorescence activated cell sorter
FCS: Fetal cattle serum
HD: Healthy donor
HDM: House dust mite
ICAM-1: Intercellular adhesion molecule 1
IFN-γ: Interferon γ
Ig: Immunoglobulin
IL-4: Interleukin-4
IL-9: Interleukin-9
ILC: Innate lymphoid cell
MFI: Mean fluorescence intensity
OVA: Ovalbumin
PBMC: Peripheral blood mononuclear cell
SEM: Standard error of the mean
TGF-β: Transforming growth factor beta
Th cell: T helper cell
VCAM-1: Vascular cell adhesion protein 1
